# Giving a leg up part 2: the ethical challenges of Body Integrity Dysphoria

**DOI:** 10.1192/j.eurpsy.2022.287

**Published:** 2022-09-01

**Authors:** G. Dumais-Lévesque, S. Pham Thi-Desmarteau

**Affiliations:** 1 Université Laval, Département De Psychiatrie Et Neurosciences, Québec, Canada; 2 Hôpital de l’Enfant-Jésus, Consultation-liaison Psychiatry, Quebec, Canada

**Keywords:** Rare condition, Body integrity dysphoria, Ethics, Consultation-Liaison psychiatry

## Abstract

**Introduction:**

Body Integrity Dysphoria (BID) is a diagnosis, newly described in ICD-11, “characterised by an intense and persistent desire to become physically disabled in a significant way… accompanied by persistent discomfort, or intense feelings of inappropriateness concerning current non-disabled body configuration”. Patients with BID may request the amputation of healthy limbs but this raises multiple ethical challenges.

**Objectives:**

By the end of the presentation, participants 1) will better understand the new diagnosis of Body Integrity Dysphoria; 2) will be able to have some landmarks to evaluate and manage this rare condition 3) will discern the ethical challenges raised by an elective or emergent amputation request.

**Methods:**

We present a complex case we faced in Quebec City, Canada. A young adult admitted to the intensive care and burn unit was referred to our Consultation Liaison (CL) team. For the second time in a year, the individual deliberately burned his leg, with the intention of having an amputation. Based on the available literature and our experience, we explore the ethical aspects of this case.

**Results:**

For this situation, the multidisciplinary team faced uncertainty and ambivalence toward the best treatment options. Deontological concerns and ethical issues emerged from the patient’s request for amputation.

**Conclusions:**

We outline how ethical concepts helped us to gain a shared comprehension of the patient’s extraordinary request, both during treatment and afterwards.

**Disclosure:**

No significant relationships.

